# Asian Primate Species Richness Correlates with Rainfall

**DOI:** 10.1371/journal.pone.0054995

**Published:** 2013-01-30

**Authors:** Yi-Chen Wang, Amrita Srivathsan, Chen-Chieh Feng, Agus Salim, Myron Shekelle

**Affiliations:** 1 Department of Geography, National University of Singapore, Singapore, Singapore; 2 Department of Biological Sciences, National University of Singapore, Singapore, Singapore; 3 Hatfield Consultants, North Vancouver, British Columbia, Canada; 4 Department of Biology, Museum of Vertebrate Biology, Portland State University, Portland, Oregon, United States of America; University of New South Wales, Australia

## Abstract

Previous studies of meta-analyses found significantly positive correlations between primate species richness and rainfall for Africa, Madagascar and the Neotropics, with the exception of Asia, leaving the open question whether that anomaly is the result of sampling bias, biogeography, or some other factor. This study re-examines the question using modelled data, with primate species richness data from the Southeast Asian Mammals Databank and rainfall data from the Climatic Research Unit. Data processing with Geographical Information Systems resulted in 390 sample points. Reduced major axis and ordinary least squares regressions were employed to examine the relationship for six regions, including the whole study area of Southeast Asia, and the subareas of Huxley West, Huxley East, Mainland Southeast Asia, Borneo, and Sumatra. The results showed a significant positive relationship between primate species richness and mean annual rainfall for Southeast Asia (r = 0.26, P<0.001). Comparing the results for the large islands and Mainland Southeast Asia showed that Sumatra had the highest correlation (r = 0.58; P<0.05). After controlling for the major biogeographic effect associated with Huxley’s Line, our results showed that primate species richness is positively associated with mean annual rainfall in Southeast Asia. Our findings contrast to prior studies of meta-analyses that showed no relationship between rainfall and primate species richness in Asia, and thereby bring Asia into agreement with results showing significant positive correlations between rainfall and primate species richness everywhere else in the world. The inference is that previous anomalous results for Asia were result of sampling bias in the meta-analysis.

## Introduction

Understanding the causes of spatial variation in species richness is one of the major research goals of macroecology and biogeography [1]. Geographical factors that have been documented to influence species richness include, for example, the well recognized latitudinal gradients [2]–[5], the established species-area relationships [6]–[8], and the widely studied but highly debated productivity-biodiversity relationship [9]–[13]. Numerous studies have examined the broad geographical factors underlying species diversity of many groups of vertebrates, especially for birds [3], [14]–[16]. In contrast, although primates are among the most intensively studied mammals, relatively few attempts have explored the factors affecting primate species richness at the regional or continental level.

Non-human primates are distributed over Asia, Africa, Madagascar, and the Neotropics. Despite this wide distribution, around 90% of primate species are concentrated within tropical rain forests [17]. This argues for the importance of factors, such as climate and forest biome, in primate species distribution [18]. Indeed, past research has reported primate species richness as a function of rainfall [19], [20] and plant productivity [21].

One curious and widely disseminated feature of previous studies, however, is that the results for Asia are anomalous, with respect to other regions (see [20]). Comparative analysis of primate species richness in over 70 sites obtained from the published literature in Asia, Africa, Madagascar, and the Neotropics showed a significantly positive correlation between the number of primate species and the mean annual rainfall within all regions, with the exception of Asia [19]. The absence of a pattern for Asia is likely because of one or more of three reasons. First, the anomalous result for Asia could be the result of Type II error caused by sampling bias, an inherent problem of meta-analyses [11]. Second, Southeast Asia is regarded as one of the most complex biogeographic regions on Earth, and it is possible that historical biogeographic factors influenced the negative result for Asia. Tropical Asia consists of numerous islands. The area and distance effects of island biogeography theory posit that small and isolated islands away from a source population tend to have low species richness in comparison with large landmasses [6]. Similarly, geologic history plays an important role in shaping the spatial variation in faunal richness. The Sunda Shelf islands to the west of Wallace’s Line were connected to Asia when sea level was lower during the Pleistocene Epoch, whereas no land bridge connected Asia to islands east of Wallace’s Line, e.g. Sulawesi, Lombok, etc. [22]. Consequently, there are stark differences in mammals across Wallace’s Line [23]. Third, anyone of a variety of other factors could have caused the negative result for Asia. For example, the monsoonal climate produces marked dry and wet seasons in many parts of tropical Asia. The extreme seasonal variation in rainfall might have accounted for a lack of relationship between rainfall and species diversity [20], [24], [25], and this would require investigation should the negative result for Asia be confirmed by subsequent studies. Thus, a central objective for advancing studies of species richness is to ascertain the underlying cause of the negative result for primates in Asia.

Another issue in the debate concerns the nature of the relationship. Kay et al. [21] revealed a unimodal relationship between primate species richness and annual rainfall in the Neotropics, peaking at 2500 mm and then declining. They also found a similar pattern for Asia by re-analysing Reed and Fleagle’s [19] data. When plotting plant productivity as a function of rainfall, the pattern mirrored the distribution of primate species richness, and based on this linkage, they suggested that plant productivity plays a role in species richness of Neotropical primates. However, it has been noted that the non-linear relationship maybe overemphasized because of the presence of very few samples with low species richness at high rainfall [26].

All of these previous analyses were dependent, partially or wholly, on literature searches. They therefore suffered from the issues related to meta-analyses in that research designs varied from study to study, and the aims of the original studies were often profoundly different from the meta-analyses [11]. Moreover, idiosyncrasies in the sampling of literature would affect the results. To illustrate this point, in the previous analyses (e.g. [19], [20]), Asia was represented by an idiosyncratic collection of sample points from the literature, such that all but one point came from regions of rainfall above 1500 mm annually. This collection of sample points would have influenced the correlations found, because the left-hand side of the graph (i.e. regions of low rainfall) were virtually unsampled, in spite of the fact that large areas of Asia have rainfall well below 1500 mm annually. A re-examination of primate species richness and rainfall in Asia, with sampling that adequately covers the range of rainfall found within Asia, is therefore needed to better answer the question as to whether primate species richness and rainfall are correlated in Asia.

The advancement in Geographical Information Systems (GIS) technology has enabled the analysis of broad spatial patterns of primate species distribution [27]. Thus, in this study, we use spatial analysis and GIS-modelled data to examine the relationship between primate species richness and mean annual rainfall across Southeast Asia, the region of Asia for which the comprehensive primate distribution data are available. Our research revisited the anomaly of the relationship between primate species richness and rainfall for Asia in two seminal publications in the *Proceedings of the National Academy of Sciences USA* (i.e. [19], [21]) and one standard textbook (i.e. [20]), which together have been cited more than 1500 times according to Google Scholar. We used modelled data to test whether the anomaly holds, or if it might be the result of sampling error from the meta-analysis, which obscure the biogeographical effect. The study aimed at filling the research gap in the knowledge of the relationship between primate species richness and rainfall in Asia. Specially, we address three questions. First, is primate species richness statistically significantly correlated with mean annual rainfall in Southeast Asia? Second, are the patterns of correlation different for the biogeographical regions on the two sides of the Wallace’s Line? Third, are the patterns of correlation different between mainland and large islands?

## Materials and Methods

### Study Area

The study area lies between 11°S and 28°N and 92°E and 128°E. The region includes continental and insular Southeast Asia. The continental Southeast Asia comprises Myanmar, Thailand, Lao People’s Democratic Republic, Cambodia, Vietnam, and Peninsular Malaysia. It experiences greater seasonality, more extremes in both temperature and rainfall, and a more pronounced dry season. The insular Southeast Asia includes the Philippines, East Malaysia (the states of Sarawak and Sabah), Brunei, Singapore, Indonesia, and East Timor, most of which experience humid equatorial climate [28].

**Figure 1:**
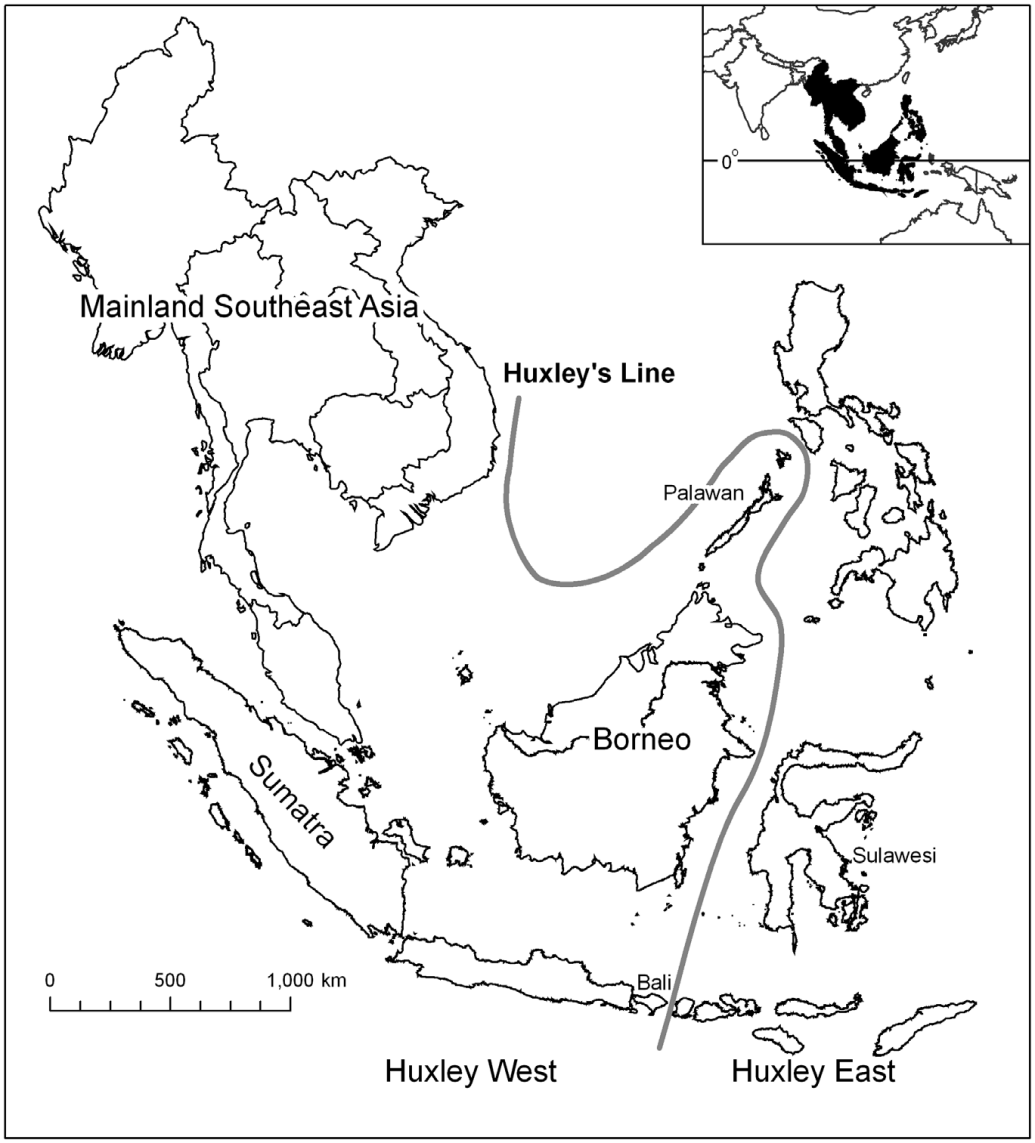
The study area of Southeast Asia and its subdivisions for analysis. The solid gray line is Huxley’s Line that separates the whole study area into the regions of Huxley East and Huxley West. Within the region of Huxley West, three regions are further analysed, including Mainland Southeast Asia, Borneo, and Sumatra.

The region has an important faunal boundary based on the biogeographical work of Wallace (i.e. Wallace’s Line, coined by T. H. Huxley in 1868). There have been a number of variants of Wallace’s Line [29]. We employed Huxley’s [30] modification (hereafter, Huxley’s Line), which relied primarily on bird and mammal distributions [31]. The line runs east of Bali, then up between Borneo and Sulawesi, and from there extending northward to include the island of Palawan to the Oriental Biogeographical Region (Fig. 1) [32]. The line also delineates the extent of Sunda Shelf. During maximum glaciations before the end of the Pleistocene (11,700 years ago), water locked up by the ice sheets caused the sea level to be some 120 m lower than the present day [22]. Borneo, Sumatra, and other islands to the west of Huxley’s Line were united with the continental Southeast Asia into an extensive contiguous landmass. Some islands to the east of Huxley’s Line (e.g. Sulawesi) were, however, separated from the mainland since the Pliocene (about 5 Ma) [32].

### Primate Species Richness and Rainfall Data

There are various ways to measure species richness, and the term carries different meanings in different contexts. Since we are using GIS-modelled data based upon estimated extent of occurrence of primate taxa, our definition of species richness is the number of species at a given sample point that would hypothetically be sympatric if their distribution was continuous throughout the polygons of their extents of occurrence. This is the meaning of species richness that we intend throughout the paper. This is equivalent to local species richness as employed by Kay et al. [21] and similar to that defined by Lawes and Eeley [18].

To calculate primate species richness, we used data from the Southeast Asian Mammals Databank (SAMD) at http://www.ieaitaly.org/samd/ [33]. The SAMD is a comprehensive database on distribution and ecology of more than 1000 mammal species [34]. We obtained a total of 99 GIS data layers from the SAMD, each of which represented the extent of occurrence of a primate species or subspecies. We followed the SAMD in adopting the primate taxonomy by Wilson and Reeder [35]. Using the Geoprocessing tools in ArcGIS 10 [36], we derived a single GIS polygon layer of primate species richness distribution, with the number of primate species ranging from 1 to 11 in each of the polygons.

For rainfall data, we used the Global Climate Dataset produced by the Climatic Research Unit (CRU) of University of East Anglia (i.e. the CRU TS 2.1 data set). This data set, available at http://csi.cgiar.org/cru/, includes monthly time-series of climatic variables from 1901 to 2002 in GIS point data format. The data resolution for the land surface of Southeast Asia is at 0.5 degree resolution, resulting in a total of 1288 climatic data points for the study area. For each of the 1288 data points, we calculated the mean annual rainfall using the monthly time-series data from 1901 to 2002.

### Associating Primate Species Richness with Rainfall

To examine the relationship between primate species richness and mean annual rainfall, we first overlaid the 1288 climatic data points with the derived polygon layer of primate species richness distribution in GIS. We removed polygons with no climatic points from the analysis, most of which were sliver polygons smaller than the 0.5 degree gap between the climatic data points. Sliver polygons in our analysis are likely as a result of small discrepancies when digitizing extents of primate occurrence. A total of 390 polygons remained. Each primate species richness polygon was typically associated with more than one climatic sampling point because the 1288 climatic data points were regularly distributed at 0.5 degree interval. To decrease spatial dependence, instead of associating the 1288 climatic data points with primate species richness directly, similar to Kozak and Wiens [37], we calculated mean annual rainfall using all the climatic points within a given polygon of the derived primate species richness GIS layer. As a result, our analysis for the whole of Southeast Asia had a total of 390 sample points, each of which was associated with values for primate species richness and mean annual rainfall.

With these sample points, we examined the relationship between primate species richness and rainfall for the following six regions indicated in Figure 1: (1) “Southeast Asia”, covering the whole area in the figure; (2) “Huxley West”, the region to the west of Huxley’s Line, illustrating the extent of Sunda Shelf; (3) “Huxley East”, the region to the east of Huxley’s Line, consisting of the Philippines, Sulawesi, and the Lesser Sunda Islands, except for Bali; (4) “Mainland Southeast Asia”, including continental Southeast Asia, except for their surrounding small islands; (5) the island of “Borneo”, which is occupied by the countries of Indonesia, Malaysia and Brunei; and (6) the Indonesia island “Sumatra”. We considered Borneo and Sumatra as individual regions for testing because they are the two largest islands in the study area, and their sizes, 748,168 km^2^ and 443,066 km^2^, respectively, are comparable to Madagascar (587,713 km^2^), for which Reed and Fleagle [19] found a positive correlation between primate species richness and rainfall.

We investigated if the average species richness in the two regions separated by Huxley’s Line and between mainland and island were different using Welch’s *t* test because of unequal variances. We diagnosed the normality of the rainfall data for each of the six regions using the Shapiro-Wilks test. The diagnostic tests showed that the rainfall data for the regions of Southeast Asia, Huxley West, and Mainland Southeast Asia were not normally distributed (P<0.01). Hence, we transformed the data for these three regions into logarithmic scales. Retesting of the logarithm-transformed data showed that they met the assumption of normality at P = 0.01; the logarithm-transformed data for these three regions were therefore used for analysis. For all the six regions, we used the reduced major axis (RMA) regression to examine the relationship between primate species richness and mean annual rainfall for the reason that the independent variable (i.e. the mean annual rainfall data here) was subject to measurement error. However, the ordinary least squares (OLS) regression analysis was also conducted to allow the comparison of the results with the findings for Asia in prior studies (i.e. [19]–[21]) and to provide correlation coefficient and its square (r and r^2^) because they are defined independently of the line fitting criterion and do not differ for RMA and OLS lines [38]. All of the statistical tests and analyses were carried out in R.

## Results

Of the 390 sample points across Southeast Asia, primate species richness ranged from 1 to 11 with an average of 5.7 species per locality (Table 1). The island of Borneo, on average, had the highest species richness per locality (Mean = 7.5) among the six regions analysed. The average species richness in Borneo is also statistically higher than that in Mainland Southeast Asia (t = −11.15; P<0.001). The region to the east of Huxley’s Line had much lower primate specie richness, an average of 1.7 primate species per locality (Table 1). This was significantly lower than the average species richness for the region west of Huxley’s Line, which had a mean of 5.9 species (t = 25.19, P<0.001). The mean annual rainfall was higher in the two large islands, 3183 mm and 2458 mm respectively for Borneo and Sumatra, while the region of Mainland Southeast Asia had the lowest mean annual rainfall of 2027 mm. The variation of mean annual rainfall across Mainland Southeast Asia was wide, ranging from 881 to 4841 mm (Table 1).

**Table 1:** Regional variation in rainfall and primate species richness.

Region	N	Rainfall (mm)				Species richness	
		Min	Max	Mean	SD	Mean	SD
Southeast Asia	390	881	4841	2364	743.7	5.7	2.19
Huxley West	364	881	4841	2369	758.6	5.9	1.98
Huxley East	26	1243	3238	2296	495.0	1.7	0.67
Mainland Southeast Asia	231	881	4841	2027	614.2	5.5	1.85
Borneo	83	1938	4083	3183	543.7	7.5	1.14
Sumatra	34	1724	3090	2458	322.0	6.9	0.91

N: number of sample points; SD: standard deviation.

**Figure 2:**
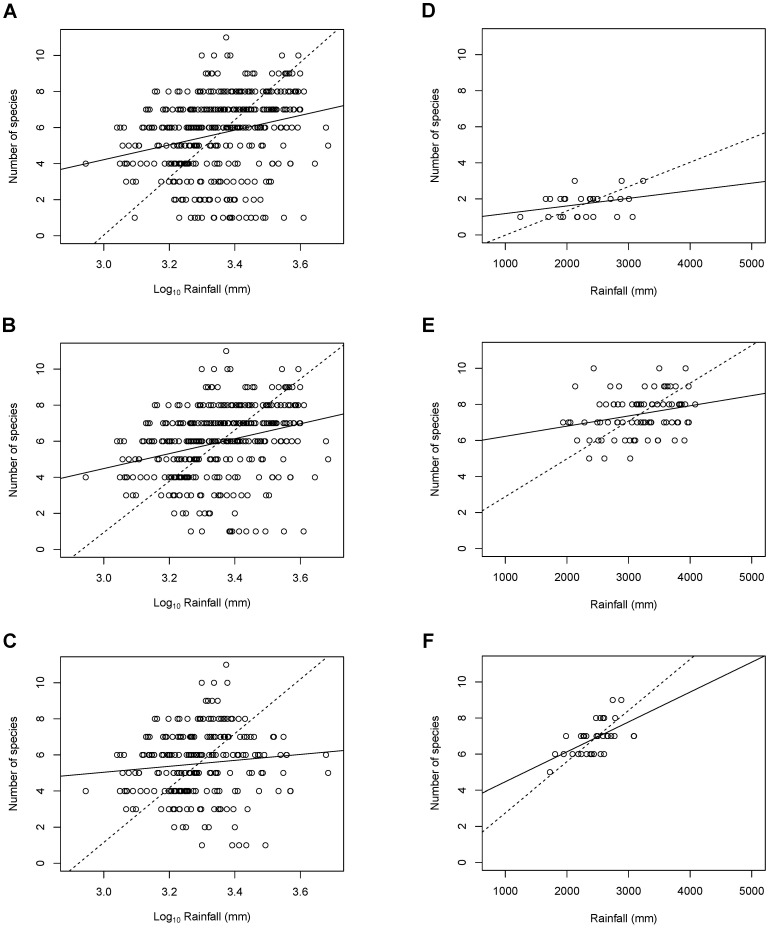
Primate species richness as a function of rainfall for (A) Southeast Asia; (B) Huxley West; (C) Mainland Southeast Asia; (D) Huxley East; (E) Borneo; and (F) Sumatra. Note that log-transformed rainfall data are used for (A) to (C) because the data of these three regions are not normally distributed. For comparison purpose, the ranges for all the y-axes are fixed; the rainfall range in the x-axis for (A) to (C) is approximately the same as that for (D) to (E) after log-transformed. The dash and solid fitted trend lines are based on the reduced major axis and the ordinary least squares regressions, respectively.

The RMA regressions showed positive relationships between primate species richness and mean annual rainfall for all the six regions analysed (Table 2, Fig. 2). The slope derived from the RMA for the whole study area Southeast Asia with logarithm-transformed rainfall data was 15.968 (95% confidence interval = 14.501, 17.582), and the results from the OLS regression for the whole Southeast Asia exhibited a significant positive relationship between rainfall and the number of primate species found (r = 0.26, P<0.001) (Table 2, Fig. 2A). When data for the region of Huxley West were analysed, the correlation between mean annual rainfall and primate species richness obtained from the OLS became slightly stronger (r = 0.29; P<0.001) (Table 2), with a slightly shallower slope of 14.191 (Fig. 2B) obtained from the RMA (95% confidence interval = 12.857, 15.664). On the other hand, in comparison with Huxley West, the correlation for the region of Huxley East was higher (r = 0.31) but not statistically significant (P = 0.119). In Borneo and Sumatra, primate species richness also displayed a significant positive linear relationship with rainfall (P<0.05) (Table 2, Figs. 2E–2F). In addition, Sumatra had the highest correlation (r = 0.58) among the six regions analysed, and a steeper slope than Borneo (Table 2). The slope derived from the RMA for the Mainland Southeast Asia was 15.069 (95% confidence interval = 13.244, 17.145) (Fig. 2C), but the region had the lowest correlation (r = 0.11) than any other region analysed, and the relationship was not statistically significant (P = 0.098) (Table 2).

**Table 2:** Slope and intercept parameters of the relationships between primate species richness and mean annual rainfall obtained from the reduced major axis (RMA) and the ordinary least squares (OLS) regressions for the six regions analysed in the study.

Region	RMA		OLS				
	Intercept	Slope	Intercept	Slope	r	r^2^	P value
Southeast Asia^a^	−47.869	15.968	−8.068	4.095	0.26	0.07	0.000
Huxley West^a^	−41.634	14.191	−7.931	4.138	0.29	0.09	0.000
Mainland Southeast Asia^a^	−44.053	15.069	0.094	1.647	0.11	0.01	0.098
Borneo	0.783	0.002	5.660	0.001	0.27	0.07	0.014
Sumatra	−0.091	0.003	2.808	0.002	0.58	0.34	0.000
Huxley East	−1.362	0.001	0.762	0.000	0.31	0.10	0.119

The correlation coefficient and its square (r and r^2^) are defined independently of the line fitting criterion and do not differ for RMA and OLS lines [37]. The P values indicate whether the relationships obtained from the OLS regressions are statistically significant.

a Logarithm-transformed data used.

## Discussion

### Primate Species Richness Correlates with Rainfall in Southeast Asia

The results of the study show positive relationships between mean annual rainfall and primate species richness for different regions in Southeast Asia (Fig. 2). Similar to the findings found in Reed and Fleagle [19] for other tropical areas such as the Neotropics and Africa that primate species richness is positively associated with mean annual rainfall, the relationship for the whole Southeast Asia exhibits a positive slope (Fig. 2A) and a statistically significant correlation between the variables (P<0.001). Contrary to the fairly strong correlations found for the Neotropics (r^2^ = 0.67), Africa (r^2^ = 0.75), and Madagascar (r^2^ = 0.70) in Reed and Fleagle [19], the highest correlation in the Southeast Asian region was found in the large island Sumatra (r^2^ = 0.34), and the correlation for the whole Southeast Asia, albeit statistically significant, was not strong (r^2^ = 0.07) (Table 2).

The results of the study contrast to prior studies that show no relationship between rainfall and primate species in Asia [19], [39]. The differences in the findings may result from variations in sample sizes, as a more extensive data set covering a wide spatial extent should allow a better evaluation of the correlation and the possible influences of biogeographical regions and island area. Reed and Fleagle [19] included 11 localities from the literature for tropical Asia; Hassel-Finnegan [39] acquired published and unpublished data from 45 protected areas in southern China, continental Southeast Asia, Borneo and Sumatra, all of which were located in the west of Huxley’s Line; our study analysed 390 points from both the Huxley West and the Huxley East regions, covering the largest spatial extent. Our data set thus extensively included localities across a wide range of rainfall from both the continental and insular Southeast Asia (Fig. 1), and from islands of both relatively low species richness (e.g. the Philippines and Sulawesi) and high species richness (e.g. Borneo) (Table 1).

### Variations in Patterns of Relations in Southeast Asia

#### Between the biogeographical regions of huxley west and huxley east

Our results indicate a major biogeographical effect associated with Huxley’s Line, which separates Bali from Lombok, Borneo from Sulawesi, and Palawan from the rest of the Philippine archipelago (Fig. 1). Compared to the Huxley West, there is an abrupt and significant decline (P<0.001) in the average number of primate species for the Huxley East (Table 1) with the number of primate species per locality ranging only from 1 to 3. Huxley’s Line represents a sharp boundary in the distribution of some mammals, and has been recognized as being an artifact of a dispersal barrier separating historical mainland Asia from lands to the east [40], [41]. During the Pleistocene Epoch, the islands in the Huxley East remained unconnected to mainland Asia at the maximum sea-level lowering at –120 m. Although Borneo and Sulawesi were closer in distance during the Pleistocene, they were still separated by a deep trench and no land bridge formed between them [23]. Hypotheses for the presence of primates to the east of Huxley’s Line in the Philippines, Sulawesi, and the Lesser Sunda Islands included: arrival by some form of sweepstakes dispersal that created a seemingly depauperate fauna [23], or even that they were transported there by humans [42]. Excluding data points of the Huxley East from the analysis increased the correlation between primate species richness and rainfall (Table 2), because it removed low species richness areas that were the result of the depauperate fauna created by sweepstakes dispersal, as opposed to being low because of low rainfall.

#### Between mainland and large islands

A concern with regard to the analysis for the region of Huxley West would be that it comprises of both mainland and islands and thus the results may be influenced by the species-area effects of island biogeography. Indeed, previous studies have suggested that the lack of relation between rainfall and number of primates at Asian site may partly reflect the fact that the continent of Asia includes many separate islands [19], [20], [39]. The sizes of islands could place an upper limit on the potential primate diversity at many sites, resulting in relatively low species diversity, regardless of rainfall [19]. However, Hassel-Finnegan [39] found that island areas did not limit species diversity in the region; primate species richness was higher for island than mainland sites, although the difference was not statistically significant. Likewise, our results showed that the mean primate species richness was statistically higher in the large islands of Borneo and Sumatra than in Mainland Southeast Asia (P<0.001).

The pattern of relation between primate species richness and rainfall for Borneo and Sumatra exhibited a significant positive relationship (Figs. 2E–2F), which was similar to that found for the large island of Madagascar [19]. Nevertheless, the relationship for the two largest islands, Borneo and Sumatra, in Southeast Asia (r^2^ = 0.07 and 0.34, respectively; Table 2) was not as strong as that for Madagascar (r^2^ = 0.70) in Africa. Unlike Madagascar of which several sites also documented low number of primate species with low rainfall data less than 1000 mm (cf. [19]), all the localities analysed in this study for Borneo and Sumatra recorded at least five or more primate species with rainfall above 1700 mm (Figs. 2E–2F; Table 1). In addition, the difference in the strength of correlation between the Southeast Asian large islands and Madagascar was likely as a result of the latitudinal influence on species richness in Madagascar. Both Borneo and Sumatra straddle the equator, with the landmasses relatively balanced north and south of the equator (Fig. 1). On the contrary, Madagascar is situated between 12° and 26° South latitude, and hence, the pattern of relation between species richness and rainfall in Madagascar may be further enhanced by the latitudinal gradient.

The high average primate species richness in Borneo and Sumatra compared to other regions analysed in this study also corresponded with the high average local species richness shown in Lawes and Eeley 18. Borneo, in particular, has been found to have a higher number of endemic primate species, presumably as a result of its degree of isolation from other Sunda sub-provinces during glacial periods, compared to Sumatra that was more readily recolonized from the mainland Southeast Asia via the Malay Peninsula [22]. In concordance with this, research has shown that Borneo and Sumatra deviate by a large amount from the general pattern of the species-area relationship for islands in Southeast Asia [42]. Heaney [43] hypothesized that high species richness in Borneo and Sumatra might have resulted from their large areas allowing high levels of habitat heterogeneity for niche diversification. Our results, however, indicate that high species richness in Borneo and Sumatra deviated from the pattern of the species-area relationship for islands in Southeast Asia (cf. [42]). This might be attributed to high rainfall distributions in Borneo and Sumatra (Figs. 2E–2F).

Compared to the rainfall distribution of Mainland Southeast Asia (Fig. 2C), the rainfall distribution of the region of Huxley West (Fig. 2B) had a larger number of sample points in the mid and high rainfall range, mostly from localities of Borneo (Fig. 2E), Sumatra (Fig. 2F) and Java (data not shown). When these points were included in the analyses, the results shifted from no statistically significant correlation with primate species richness for the region of Mainland Southeast Asia, to statistically significant linear correlation for the region of Huxley West. This is particularly interesting, because previous studies have attributed the lack of relationship between primate species richness and rainfall to the presence of the large number of islands in Asia. Contrary to this, we observed that inclusion of islands into the data set increased the quantity of high rainfall sample points, thereby improving the correlation.

## Conclusions and Future Work

With a wider range of rainfall data and a larger spatial extent of examination than prior work, the results of this study indicate that primate species richness is positively correlated with rainfall in Southeast Asia. In addition, the study detects the major biogeographical effect associated with Huxley’s Line. The average number of primate species per locality is significantly lower in the Huxley East than that in the Huxley West. In comparison with Huxley West, the correlation between rainfall and primate species richness for the region of Huxley East was also not statistically significant. Within the region of Huxley West, the patterns of correlations are different between the Mainland Southeast Asia and the large islands. Primate species richness displays a statistically significant linear relationship with rainfall in large islands Borneo and Sumatra, but not in Mainland Southeast Asia. The approach employed in this study allows the re-examination of the patterns of correlation for primate species richness as a function of rainfall in Southeast Asia, enabling a better understanding of factors that govern species distribution and diversity.

That rainfall did not hold a significant relationship with primate species richness in Mainland Southeast Asia does not preclude the possibility of a non-linear correlation. Kay et al. [21] has found a non-linear unimodal relationship for the Neotropics, with primate species richness first increasing as rainfall increases but then declining gently in localities with high rainfall, as a result of decrease in plant productivity. It will be desirable to investigate if such non-linear relationship exists for the region of Mainland Southeast Asia, and whether forest productivity plays a role in primate species richness in the region. In addition, compared to prior work, the r values for the six regions analysed in Southeast Asia are low. This is not unexpected due to the larger sample sizes analyzed in the study, but also calls for attention to considering other factors as explanatory variables for primate diversity. Indeed, the present day patterns of primate species richness in Southeast Asia seem to reflect a combination of environmental and historical factors rather than a single unitary cause [20], [39]. An area for future research is to incorporate phylogeny in the analysis (e.g. [37], [44], [45]) to quantify the relative effects of space and phylogeny in simultaneously shaping the variations in species distributions and to address how the climate-diversity relationships actually arise.
